# The relationship between activation–passivation transition and grain boundary dissolution on four steel samples in acidic solutions containing NO_2_^−^

**DOI:** 10.1039/c9ra03983j

**Published:** 2019-07-30

**Authors:** Yong Zhou, Pei Zhang, Jinping Xiong, Fuan Yan

**Affiliations:** Key Laboratory for Green Chemical Process of Ministry of Education, Wuhan Institute of Technology Wuhan 430205 China zhouyong@wit.edu.cn; College of Chemistry and Food Science, Yulin Normal University Yulin 537000 China; Beijing Key Laboratory of Electrochemical Process and Technology for Materials, Beijing University of Chemical Technology Beijing 100029 China

## Abstract

Herein, for four steels (L80, N80, X65 and Q235) in acidic solutions (HNO_3_, HCl, HAc and CO_2_) containing NO_2_^−^, the relationship between the activation–passivation (A–P) transition and the grain boundary dissolution (GBD) was studied by potentiodynamic polarization curve (PPC) measurements and scanning electron microscopy (SEM) observations. In the specific pH range of acidic solutions, where the four steels showed an electrochemical characteristic of the A–P transition, GBD was observed on the steel surface; however, at low or high pH values of the acidic solutions, the four steels respectively showed the electrochemical behavior of activation (A) or self-passivation (sP), and GBD was not observed on the steel surface. The effects of the acid type, pH value and steel type on the electrochemical characteristic of the A–P transition and the occurrence of GBD were also discussed in detail. *Via* this study, it was confirmed that under the electrochemical characteristic of the A–P transition, the occurrence of GBD was a general corrosion behavior of carbon steels and alloy steels in acidic solutions containing NO_2_^−^.

## Abstract 2

The relationship between activation–passivation transition and grain boundary dissolution for L80, N80, X65 and Q235 steels in HNO_3_, HCl, HAc and CO_2_ solutions containing NO_2_^−^ was studied by electrochemical tests and microstructural observations.

## Graphical abstract

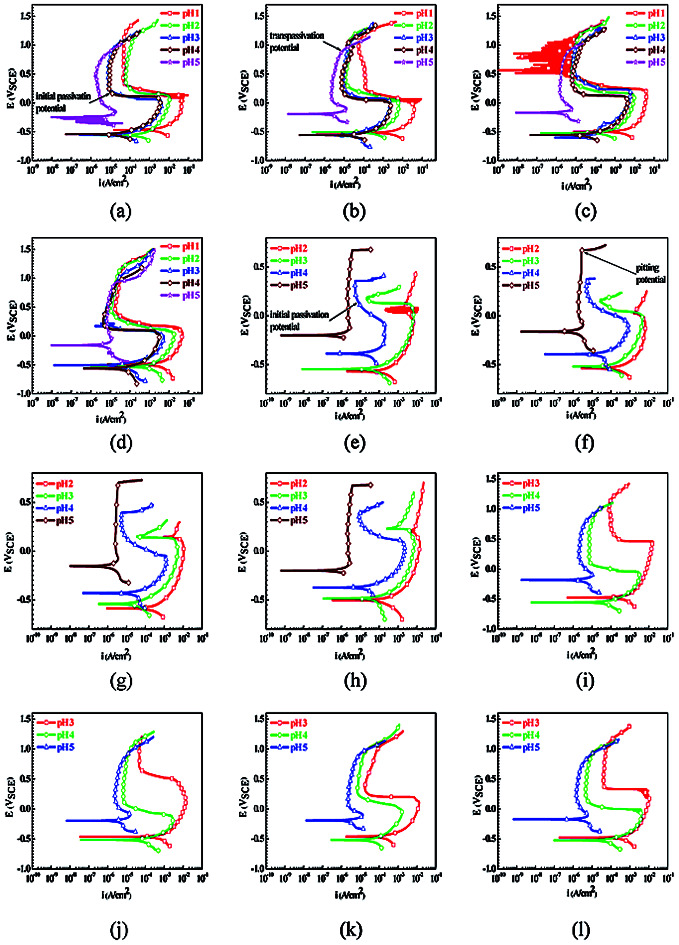

## 1. Introduction

Steel products are widely applied in the fields of production and living;^1–3^ however, the problems of corrosion and failure inevitably limit their application and development,^4–6^ which are due to the presence of aggressive species in service environments.^7–9^

In the electrochemical process of steel corrosion, the main cathodic reaction is H^+^ reduction or O_2_ reduction, which is very closely related to the solution pH value. In strongly acidic solutions (relatively low pH), the dominant cathodic reaction is the H^+^ reduction with the standard potential (*E*_st_) of −0.244 V_SCE_:^10^

1 2H^+^ + 2e → H_2_ (*E*_st_ = −0.244 V_SCE_)

According to the Nernst equation, the equilibrium potential (*E*_eq_) of H^+^ reduction can be described as follows:^11^

2 *E*_eq/SCE_ (H^+^/H_2_) = −0.244 − 0.059 pH

In contrast, in weakly acidic and alkaline solutions (relatively high pH), the O_2_ reduction with the *E*_st_ of 0.157 V_SCE_ becomes the dominant cathodic reaction:^10^

3 O_2_ + 2H_2_O + 4e → 4OH^−^ (*E*_st_ = −0.157 V_SCE_)

The *E*_eq_ of O_2_ reduction can be depicted as follows:^11^

4 *E*_eq/SCE_ (O_2_/OH^−^) = 0.984 − 0.059 pH

It is speculated that the following anodic reaction of Fe oxidation is independent of the solution pH value:

5 Fe → Fe^2+^ + 2e (*E*_st_ = −0.684 V_SCE_)

With an increase in the pH value, the *E*_eq_ for both the H^+^ reduction (eqn (2)) and the O_2_ reduction (eqn (4)) would decline; this would result in the negative shift of the corrosion potential (*E*_corr_) in the polarization curve. In addition, the left shift of the chemical equilibrium for the H^+^ reduction (eqn (1)) and the O_2_ reduction (eqn (3)) would occur, resulting in a decrease in the corrosion current density (*i*_corr_) in the polarization curve. Related studies involving the abovementioned discussion have been repeatedly reported.^12–14^

On the other hand, intergranular corrosion (IGC) is a common and important type of localized corrosion for metals and alloys, which is attributed to the establishment of an electrochemical micro-couple between grain interiors (GIs) and grain boundaries (GBs).^15^ At present, studies on IGC are mainly focused on stainless steels (SSs)^16–20^ and aluminum alloys (AAs);^21–25^ this is due to high IGC susceptibility of SSs and AAs derived from the precipitation of harmful phases along GBs during the process of inappropriate thermal treatment. For SSs, precipitates such as Cr_23_C_6_,^16^ CrN/Cr_2_N,^17^ σ phase,^18^ χ phase,^19^ and π phase^20^ have been reported to result in IGC susceptibility. Moreover, the IGC susceptibility of AAs has been reported to result from the precipitation of the θ phase,^21^ S phase,^22^ β phase,^23^ T phase,^24^ η phase^25^ and other similar phases; due to the presence of IGC susceptibility, the electrochemical micro-couple between GIs and GBs can be spontaneously established when SSs and AAs are applied in corrosive environments, thus inducing the occurrence of IGC.^26^

Except for SSs, IGC susceptibility is not prominent in other types of steel, and relatively few studies have been reported on IGC. In a previous study, for the Q235 carbon steel in a CO_2_–NaNO_2_ solution, the occurrence of the grain boundary dissolution (GBD) was observed on the steel surface when the Q235 steel was polarized in the activation–passivation (A–P) region.^27^ Subsequent studies further confirmed the occurrence of GBD on the surface of carbon steels when this type of steel was polarized in the A–P region in acidic solutions containing NO_2_^−^ such as a CO_2_–NaNO_2_ solution,^28^ HNO_3_–NaNO_2_ solution^29^ and HCl–NaNO_2_ solution.^30^ However, to date, it is not clear that in acidic solutions containing NO_2_^−^, whether the occurrence of GBD is a special corrosion behavior of carbon steels or a general corrosion behavior of other steels, particularly alloy steels. To clarify the abovementioned statement, in this study, three low alloy steels (L80, N80 and X65) were chosen to study the relationship between the electrochemical characteristic of the A–P transition and the occurrence of GBD in four acidic solutions (HNO_3_, HCl, HAc and CO_2_) containing NO_2_^−^. Electrochemical measurements using a potentiodynamic polarization curve (PPC) and microstructural observation using a scanning electron microscope (SEM) were carried out; in addition, to understand the difference between the A–P transition and GBD of carbon steel and alloy steel, the PPC measurement and the SEM observation for the Q235 steel were performed.

## 2. Experimental

The detailed chemical composition of the L80, N80, X65 and Q235 steels is listed in Table 1. Herein, four steel samples were manually abraded up to 1000 grit with SiC abrasive papers, rinsed with deionized water and degreased in acetone.

**Table 1:** Detailed chemical composition of the L80, N80, X65 and Q235 steels

Steel	C	Mn	P	S	Si	Cr	Mo	Ni	Cu	Al	Fe
L80	0.190	1.370	0.010	0.004	0.230	0.024	0.034	0.023	0.023	0.036	Balance
N80	0.240	1.280	0.015	0.015	0.310	0.015	0.026	0.026	0.015	0.020	Balance
X65	0.030	1.510	0.024	0.005	0.170	0.038	0.016	0.025	0.040	0.020	Balance
Q235	0.160	0.530	0.015	0.045	0.300	—	—	—	—	—	Balance

The tested solutions were four acidic solutions containing NO_2_^−^: HNO_3_–NaNO_2_, HCl–NaNO_2_, HAc–NaNO_2_ and CO_2_–NaNO_2_. For the first three solutions, the diluted HNO_3_, HCl and HAc solutions were introduced into a 0.01 M NaNO_2_ solution to adjust the pH value. For the CO_2_–NaNO_2_ solution, the CO_2_ gas was purged into a 0.01 M NaNO_2_ solution until the CO_2_ saturated condition was reached and the pH value of the CO_2_–NaNO_2_ solution was 3.7.^31^

The PPC tests were carried out using the CS310 electrochemical workstation. A typical three-electrode system was applied, and the system was composed of a saturated calomel electrode (SCE) as the reference electrode, a platinum sheet as the counter electrode and a steel sample as the working electrode. Before each PPC test, the working electrode was immersed in the corresponding tested solution for 30 min to ensure a stable condition of the open circuit potential (OCP). The potential scanning rate was 0.1 mV s^−1^, and the potential scanning range was from −0.3 V_OCP_ to the potential value corresponding to the occurrence of transpassivation or pitting. All the PPC tests were performed at ambient temperature. After this, the surface morphologies of the four steel samples were observed by the LEO-1450 SEM instrument.

## 3. Results and discussion

**Fig. 1:**
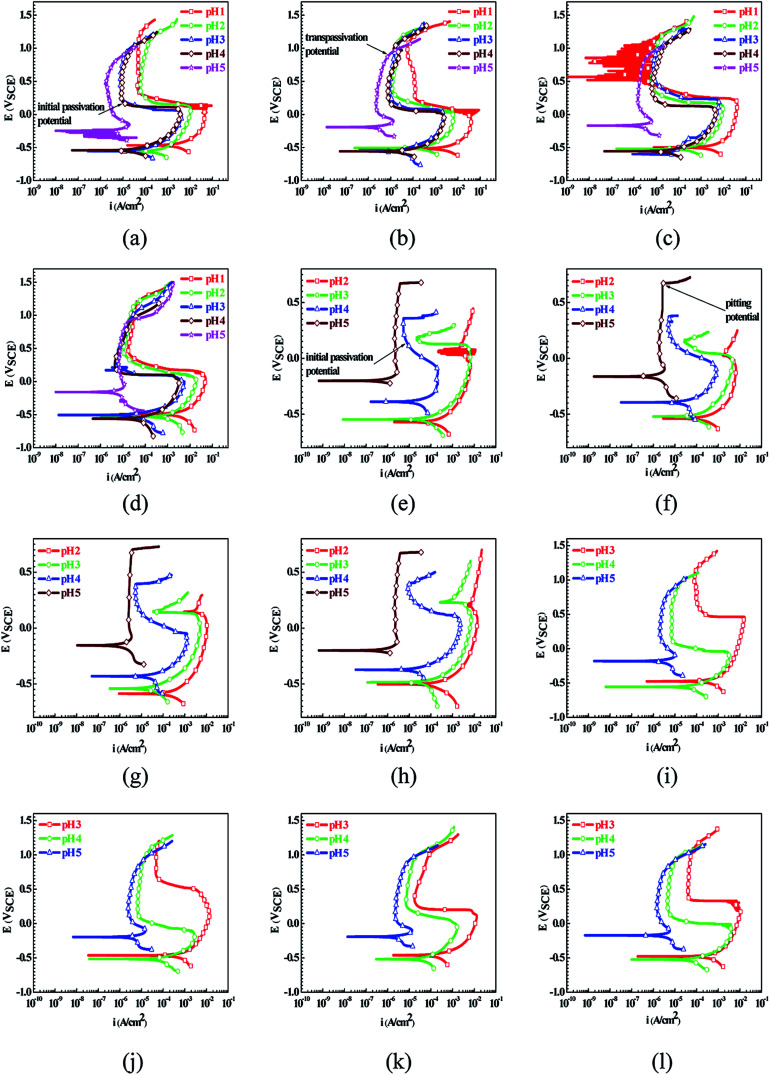
Potentiodynamic polarization curves of the L80, N80, X65 and Q235 steels in HNO_3_–NaNO_2_, HCl–NaNO_2_ and HAc–NaNO_2_ solutions with 0.01 M NaNO_2_ and different pH values: (a) L80–HNO_3_–NaNO_2_, (b) N80–HNO_3_–NaNO_2_, (c) X65–HNO_3_–NaNO_2_, (d) Q235–HNO_3_–NaNO_2_, (e) L80–HCl–NaNO_2_, (f) N80–HCl–NaNO_2_, (g) X65–HCl–NaNO_2_, (h) Q235–HCl–NaNO_2_, (i) L80–HAc–NaNO_2_, (j) N80–HAc–NaNO_2_, (k) X65–HAc–NaNO_2_ and (l) Q235–HAc–NaNO_2_.

Fig. 1 shows the PPCs of L80, N80, X65 and Q235 steels in HNO_3_–NaNO_2_, HCl–NaNO_2_ and HAc–NaNO_2_ solutions with 0.01 M NaNO_2_ and different pH values. The effects of the acid type, pH value and steel type on the electrochemical behaviors were investigated, and the following five electrochemical behaviors were demonstrated in the PPCs: activation–passivation–transpassivation (A–P–T), self-passivation–transpassivation (sP–T), activation (A), activation–passivation–pitting (A–P–P), and self-passivation–pitting (sP–P). The detailed results of the electrochemical behavior are presented in Table 2.

**Table 2:** Electrochemical behaviors and SEM morphologies of the L80, N80, X65 and Q235 steels polarized to different potential values in HNO_3_–NaNO_2_, HCl–NaNO_2_ and HAc–NaNO_2_ solutions with 0.01 M NaNO_2_ and different pH values

Acid	pH	Steel			
		L80	N80	X65	Q235
HNO_3_ + NaNO_2_	pH 1	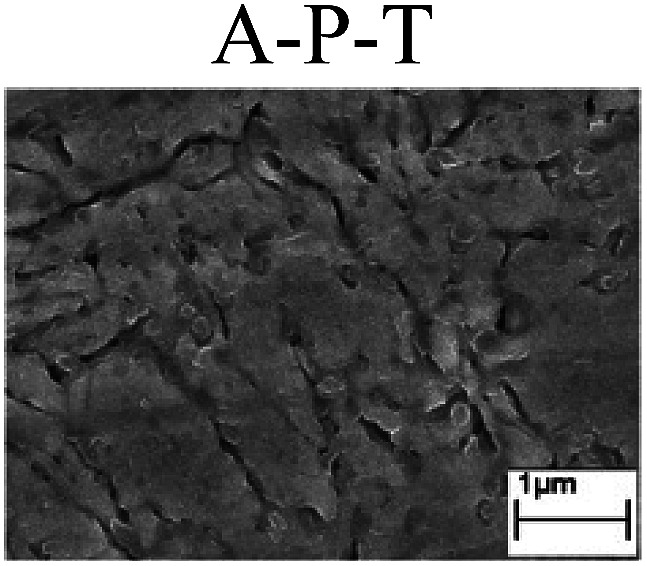	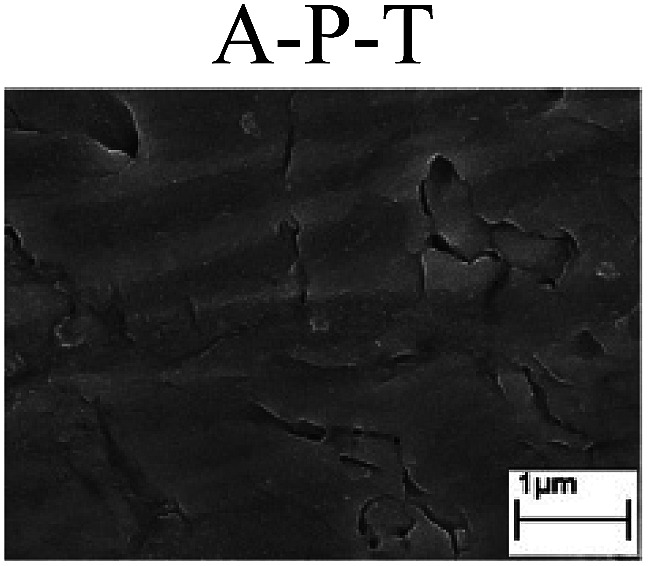	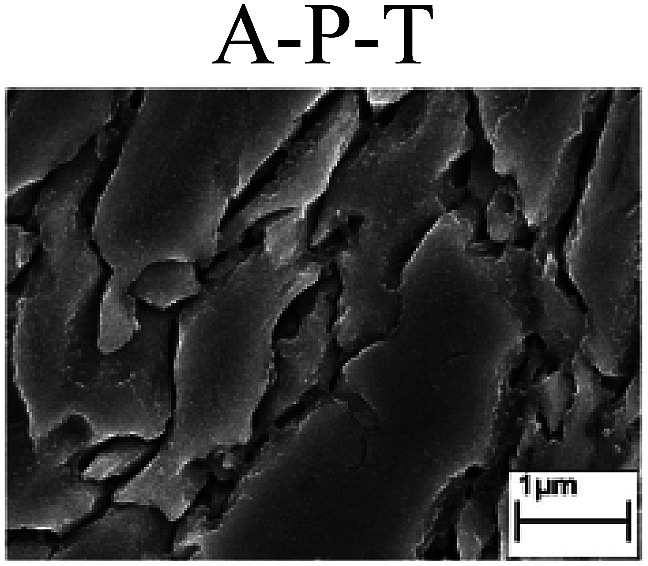	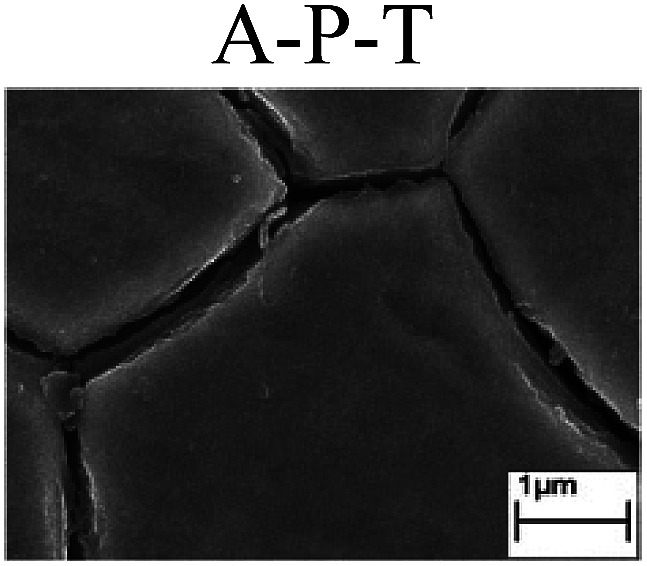
	pH 2	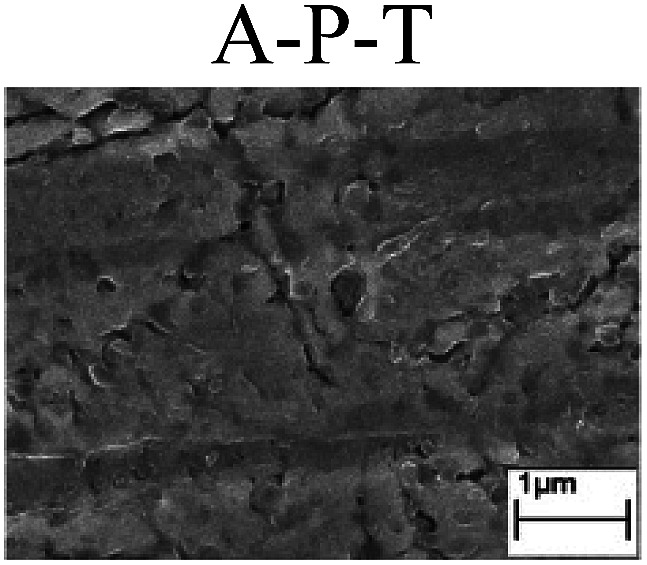	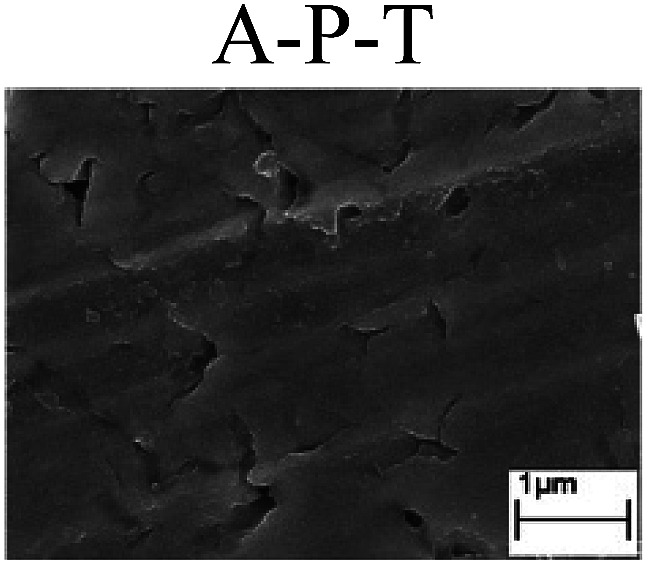	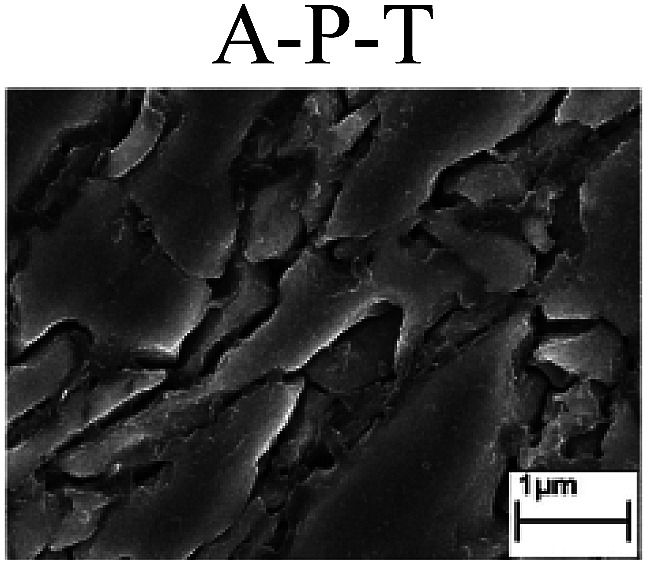	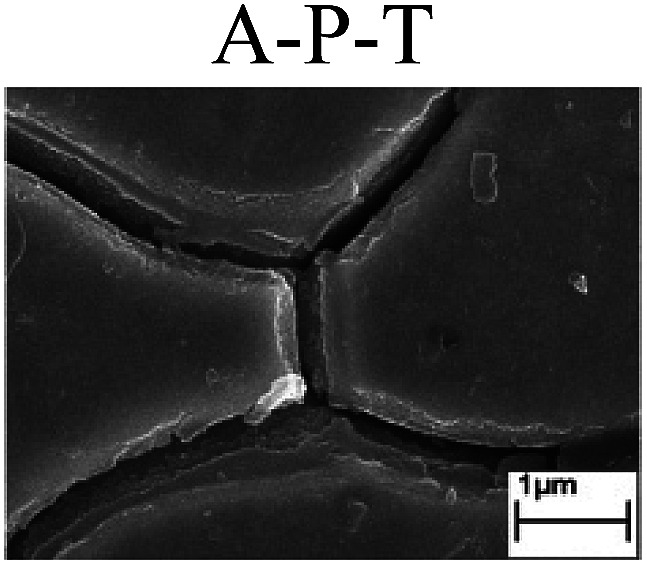
	pH 3	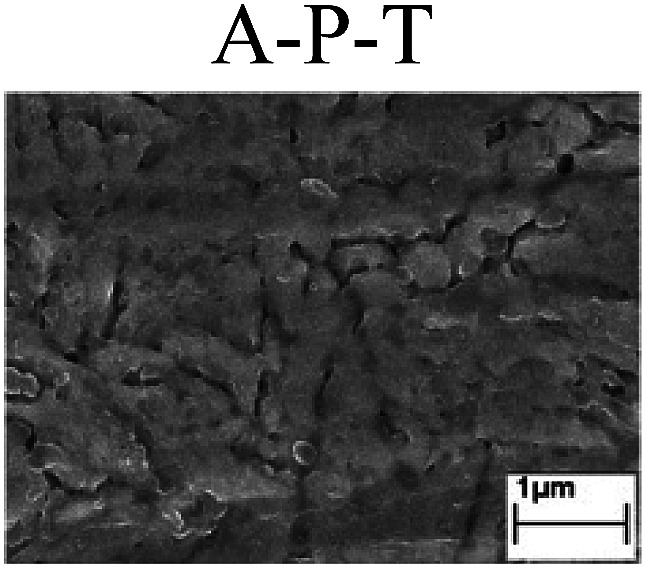	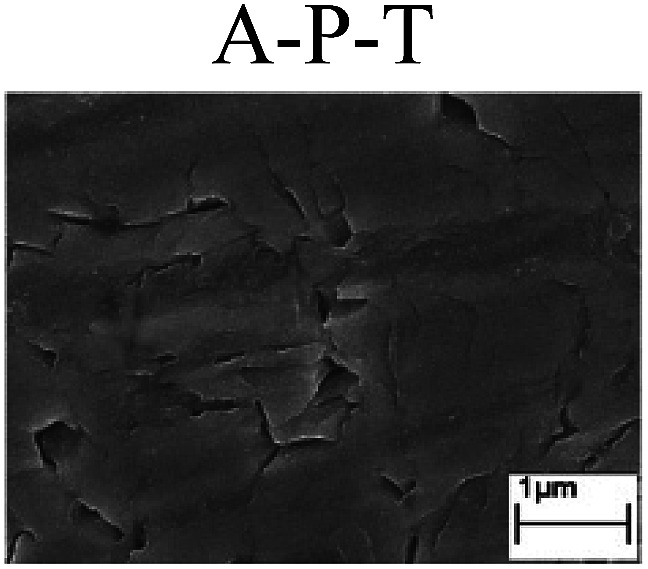	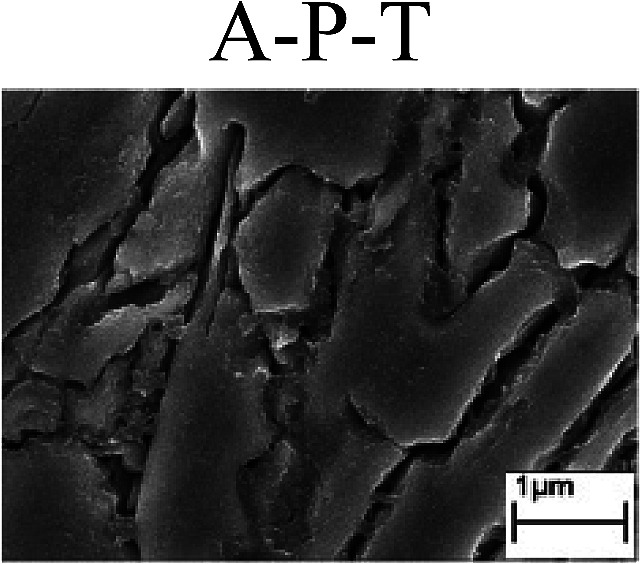	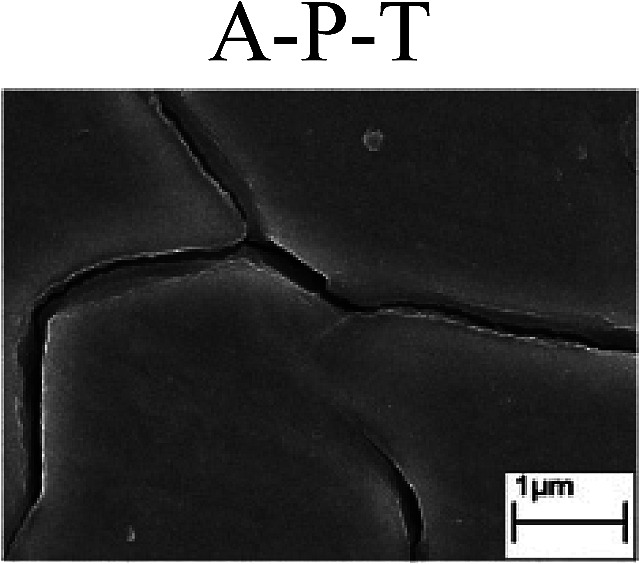
	pH 4	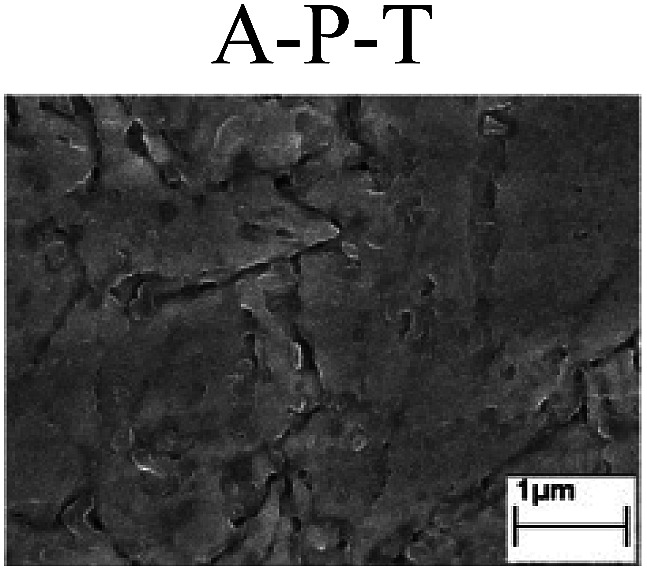	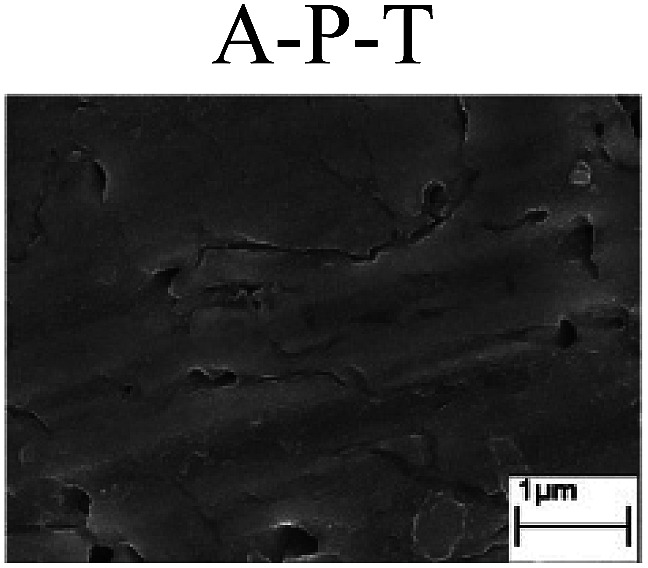	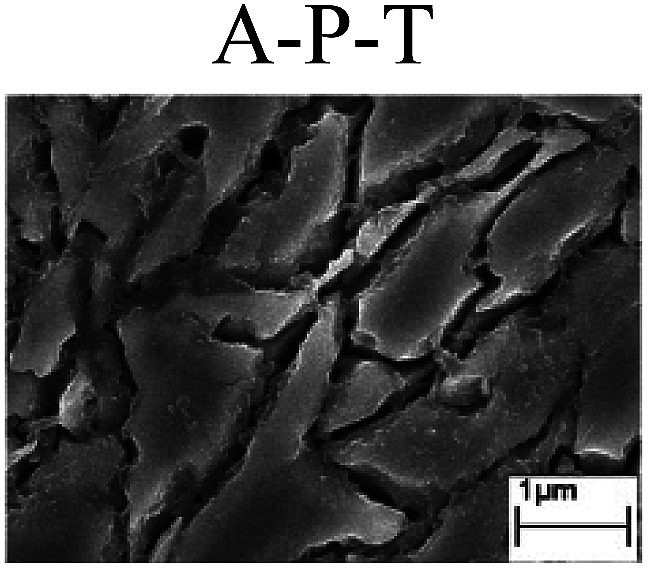	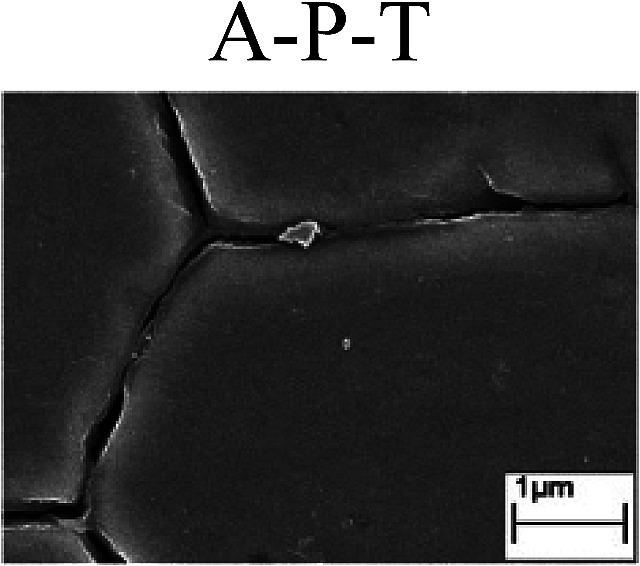
	pH 5	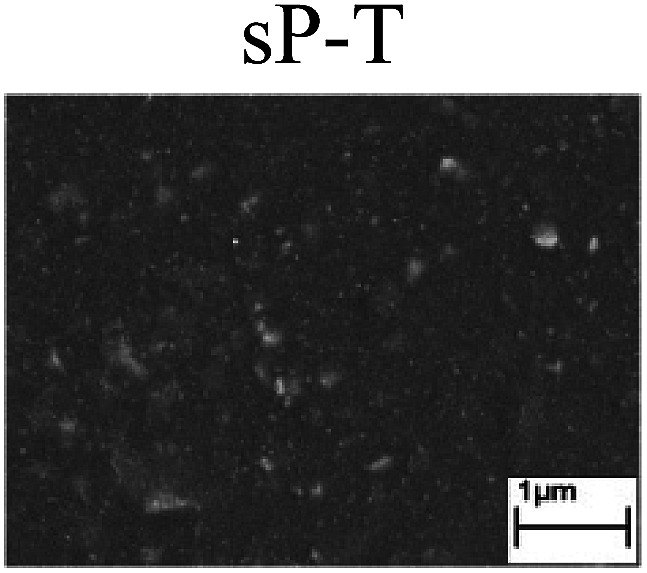	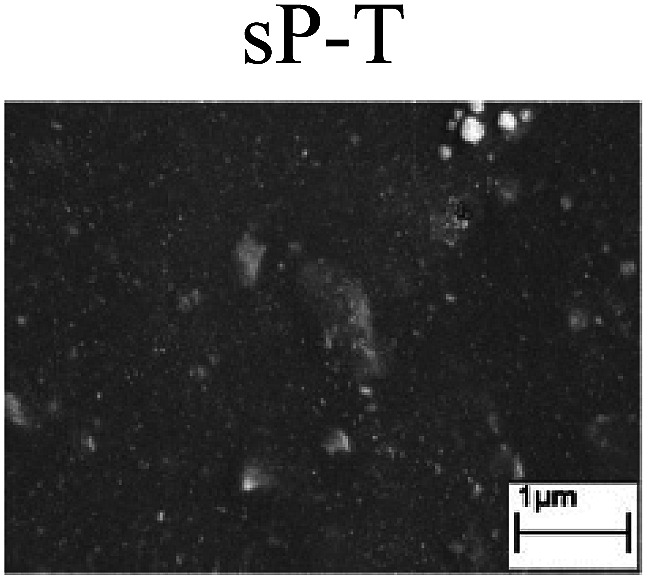	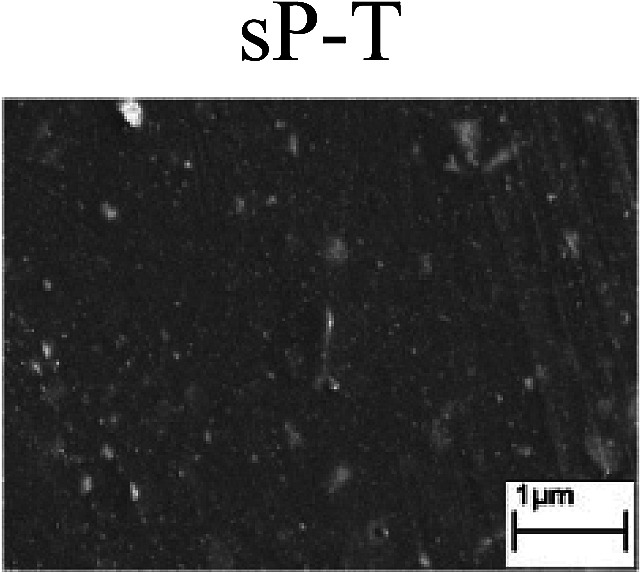	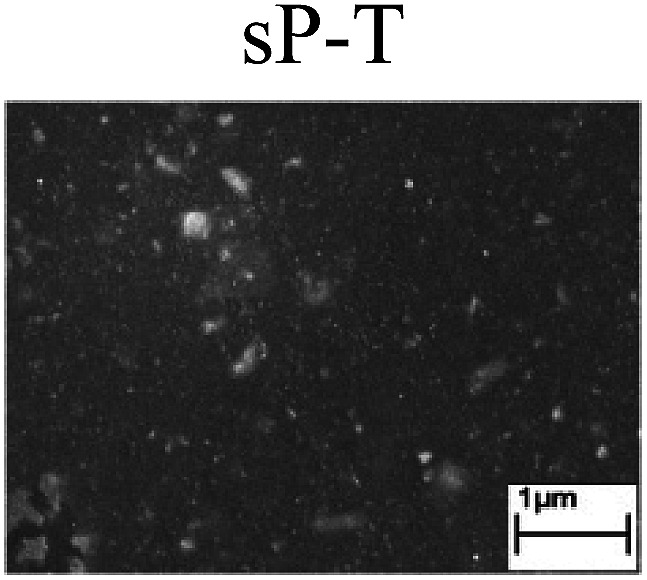
HCl + NaNO_2_	pH 2	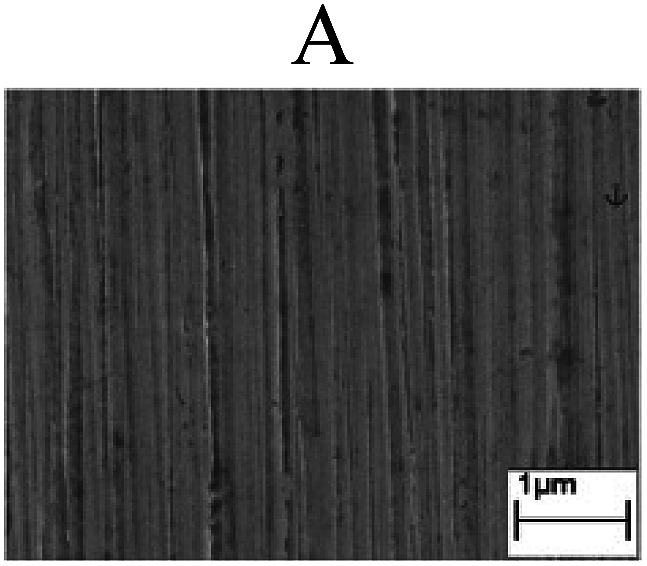	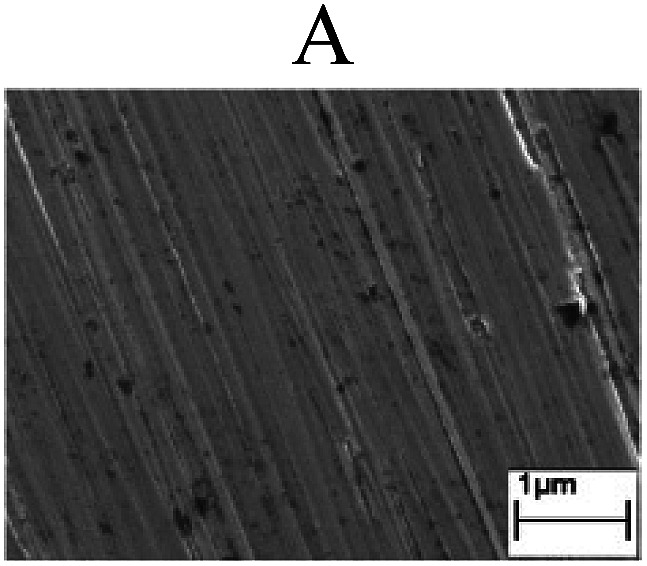	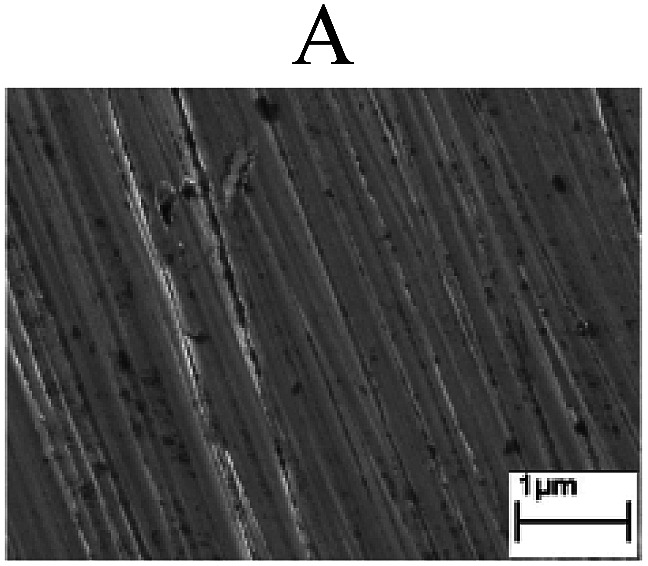	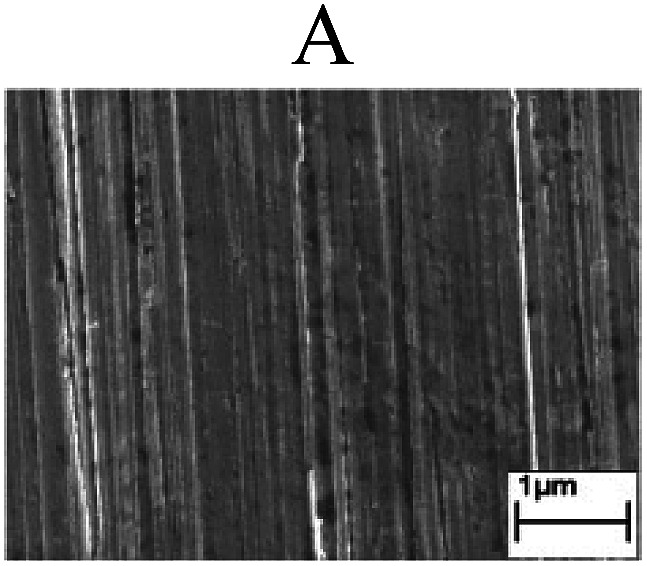
	pH 3	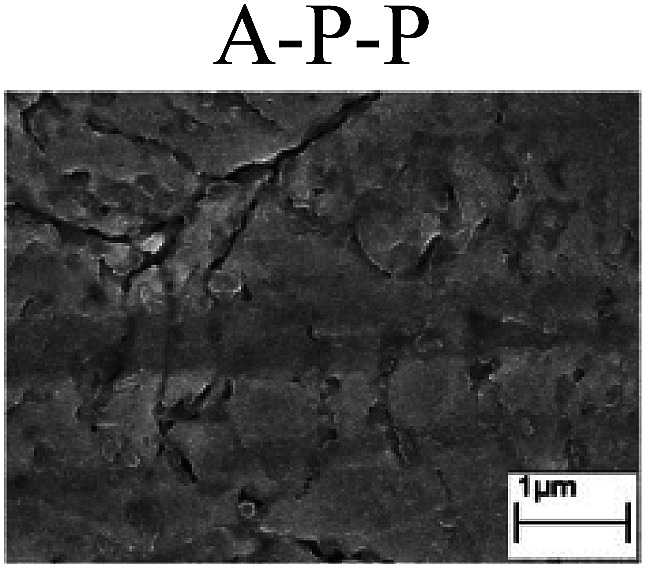	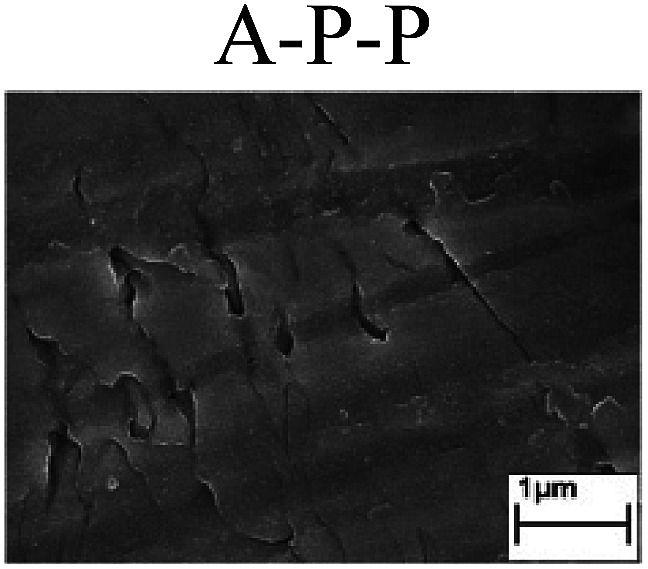	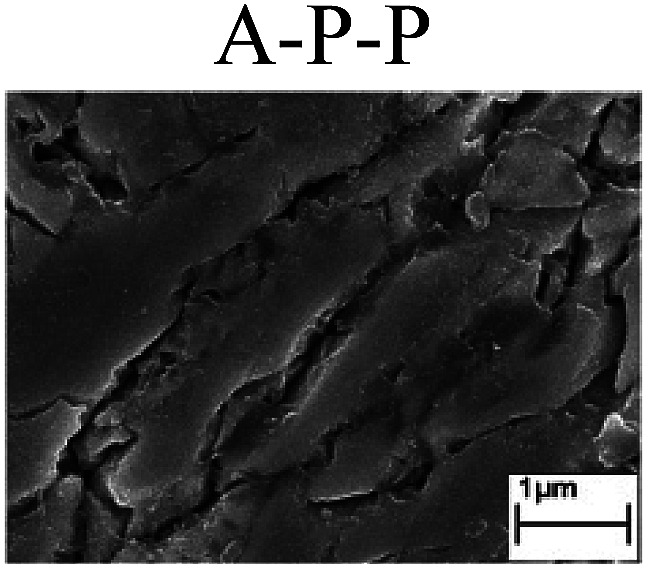	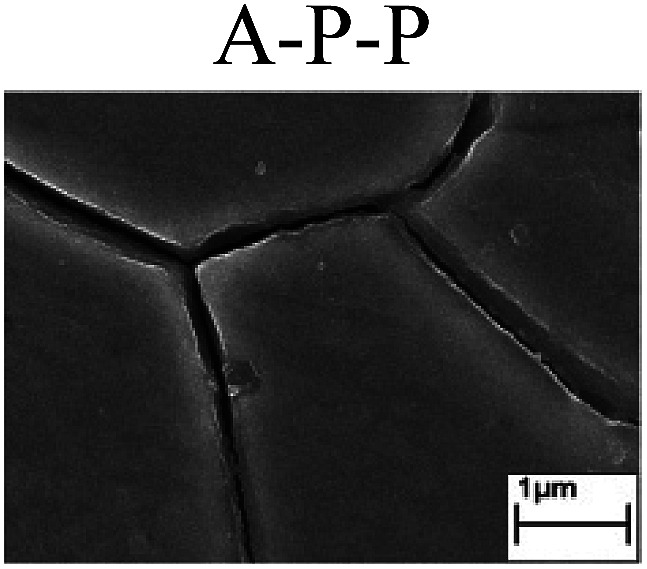
	pH 4	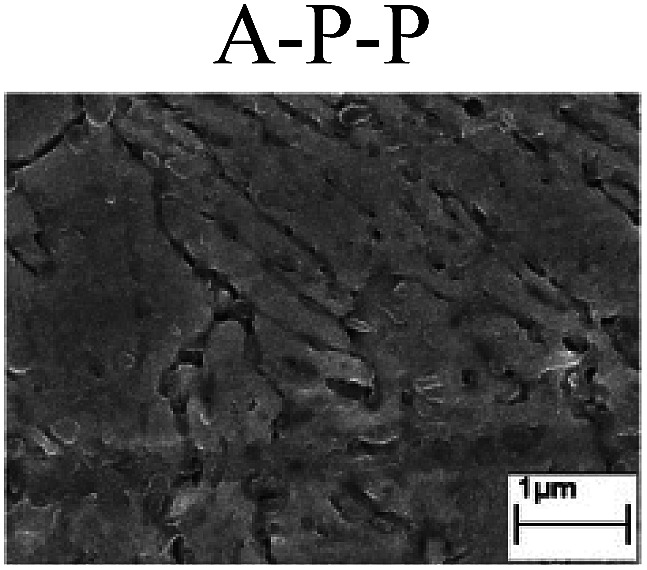	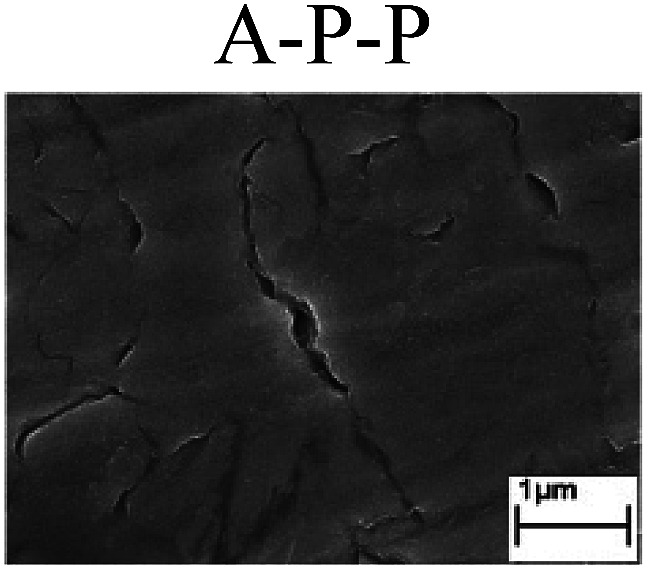	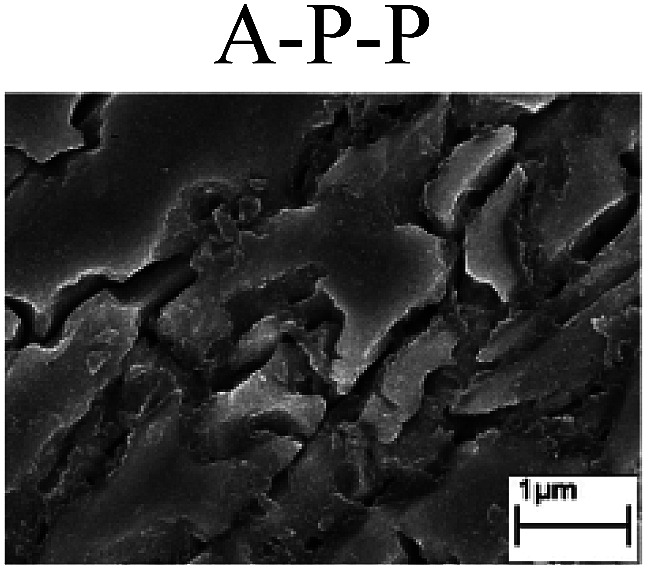	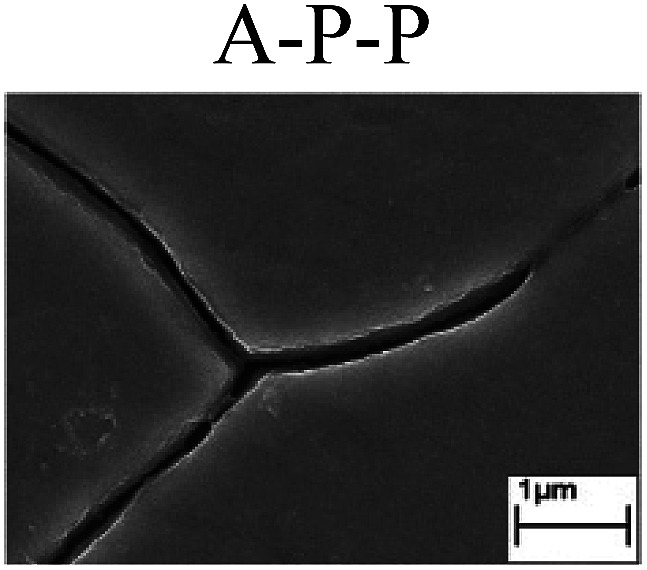
	pH 5	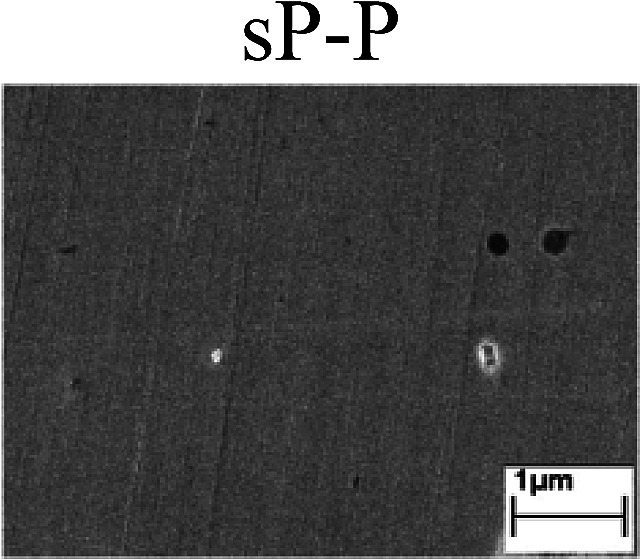	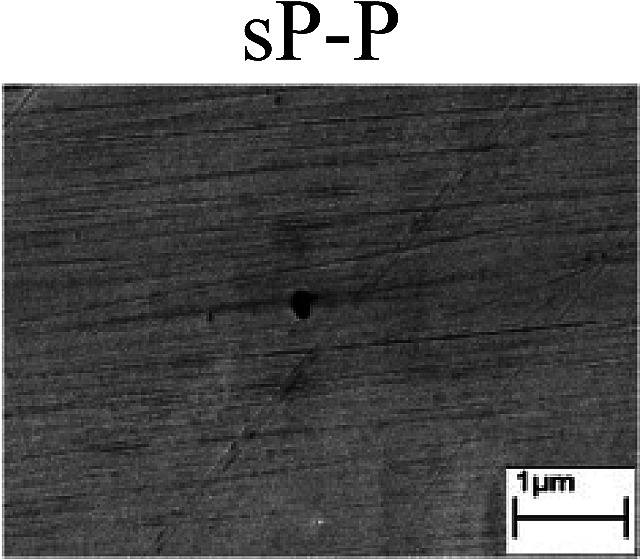	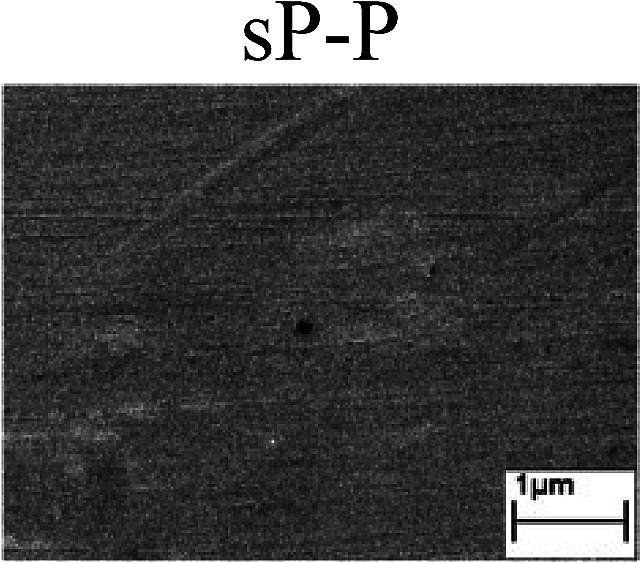	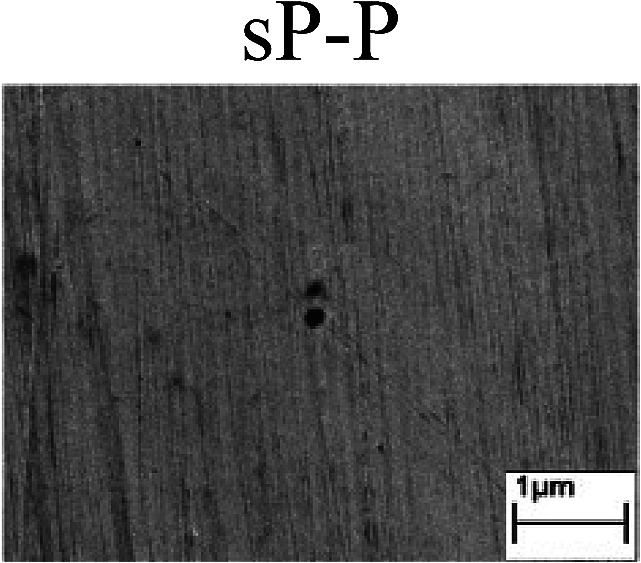
HAc + NaNO_2_	pH 3	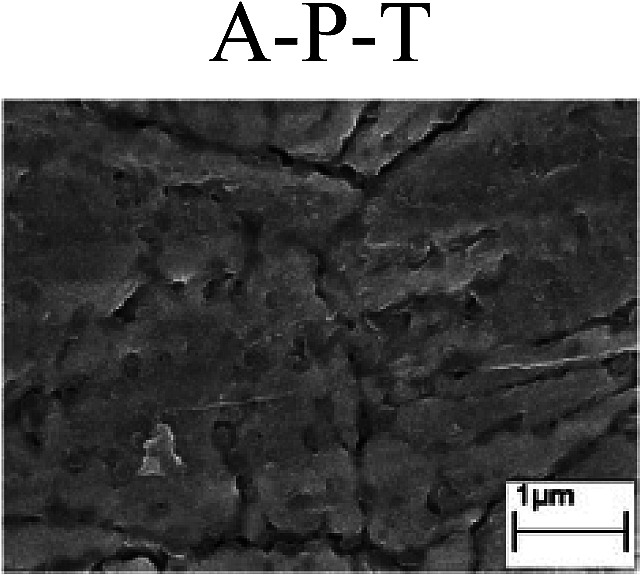	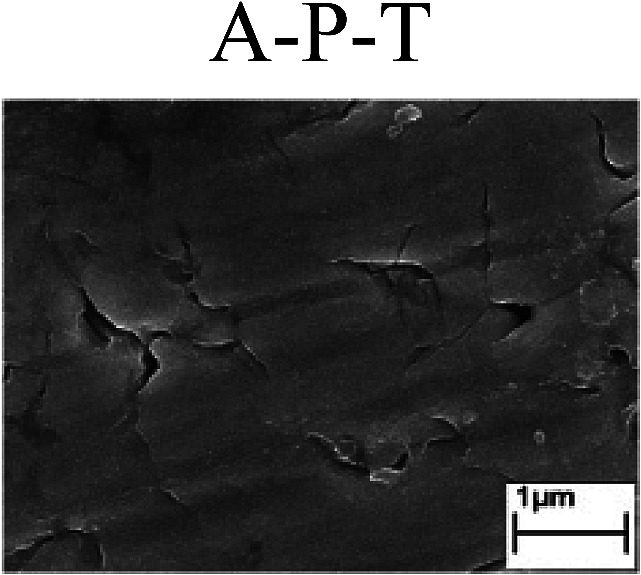	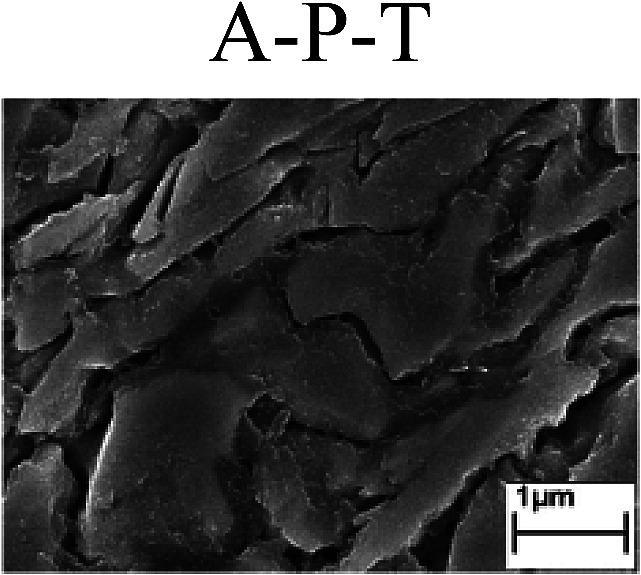	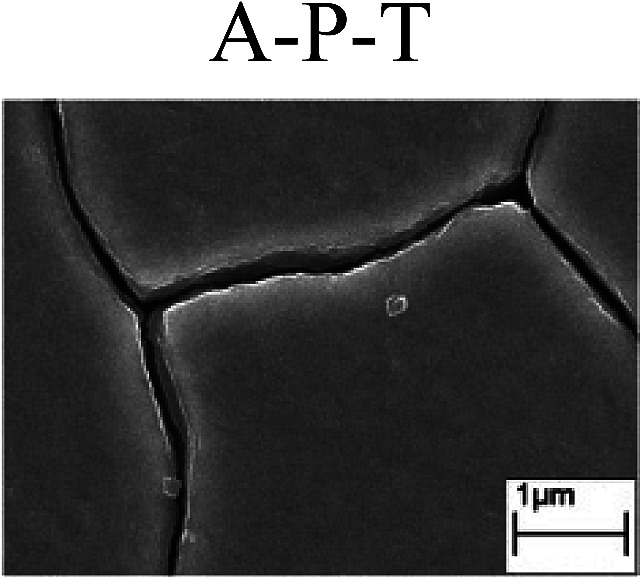
	pH4	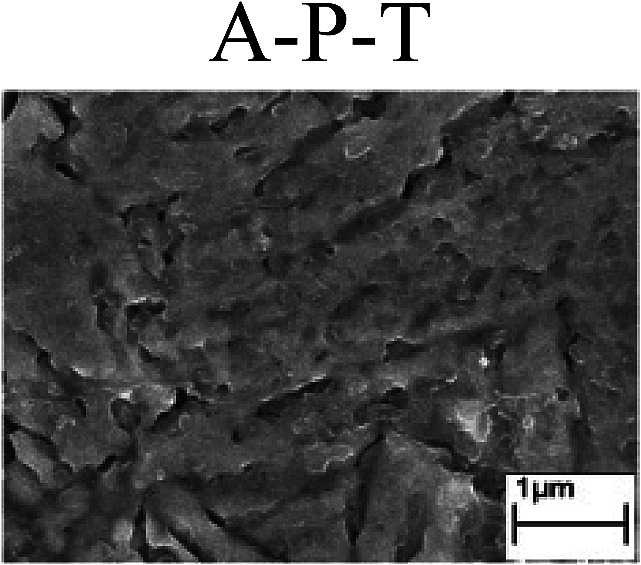	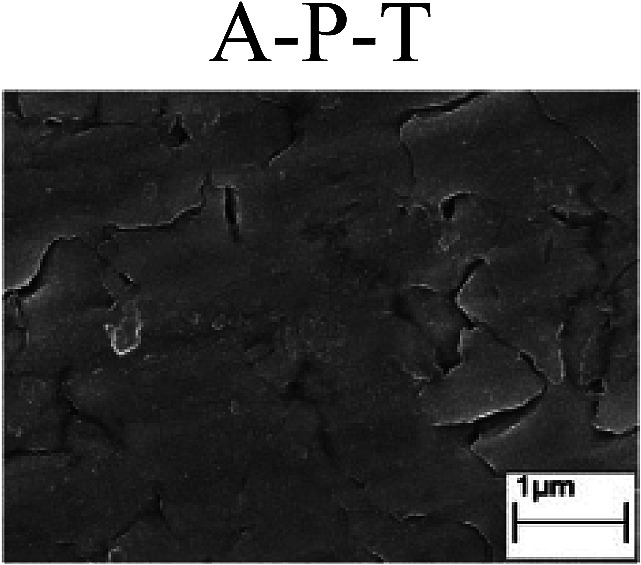	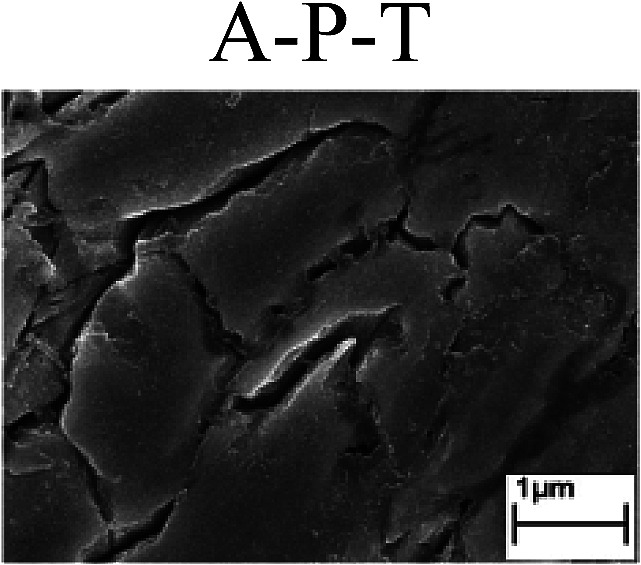	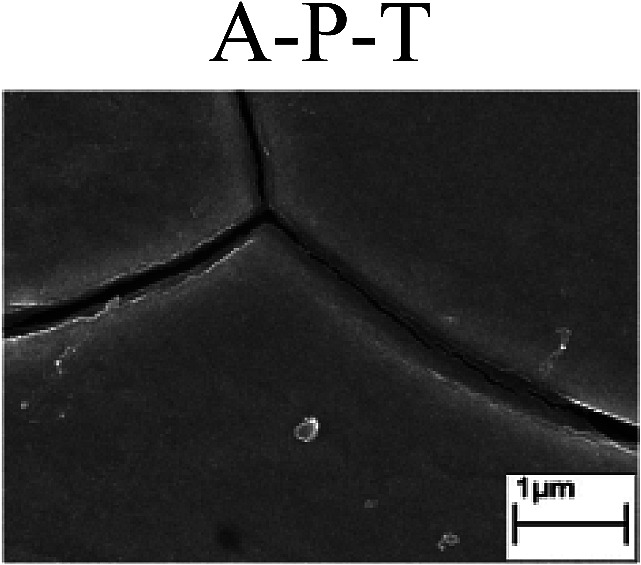
	pH 5	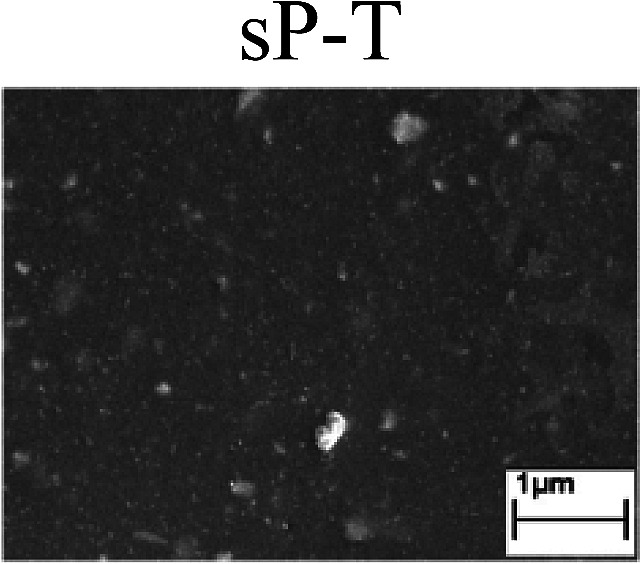	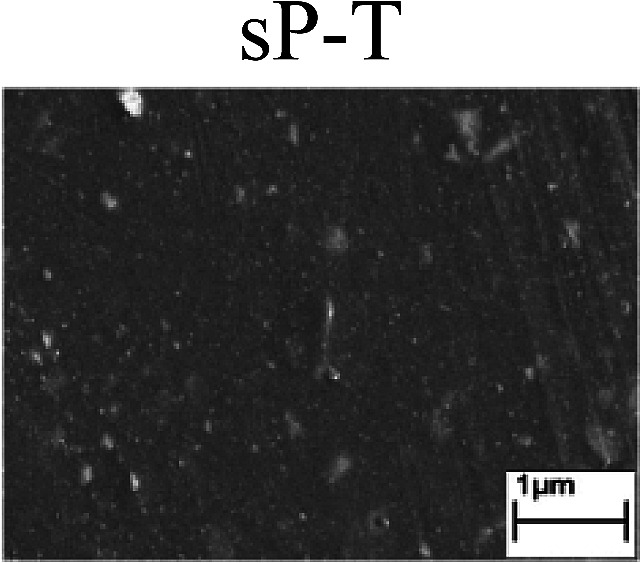	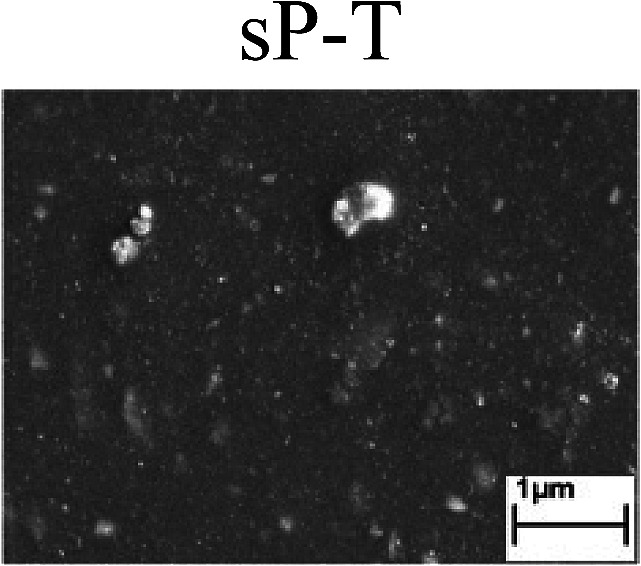	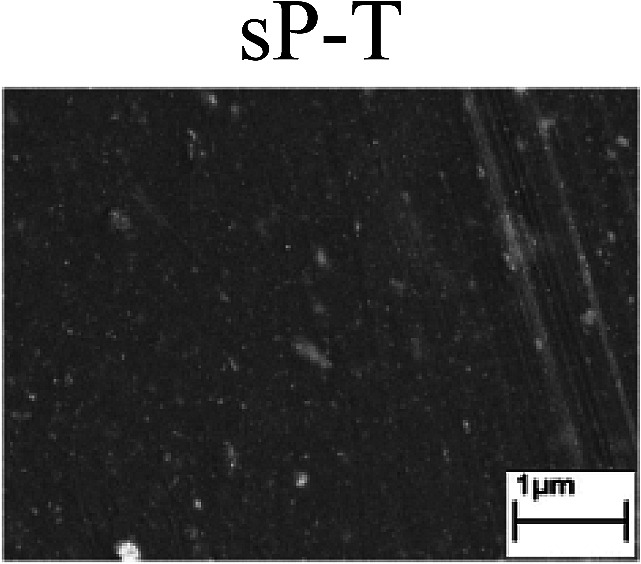

According to previous studies,^27–30^ for carbon steels in acidic solutions containing NO_2_^−^, the initiation and propagation of GBD occurs on the steel surface when the carbon steels are polarized in the A–P region. Based on the results shown in Fig. 1 and Table 2, the electrochemical characteristic of the A–P transition, including A–P–T and A–P–P, was also observed in the PPCs for the following cases: from pH 1 to pH 4 in HNO_3_–NaNO_2_ solutions, at pH 3 and pH 4 in HCl–NaNO_2_ solutions and at pH 3 and pH 4 in HAc–NaNO_2_ solutions.

To confirm the occurrence of GBD, four steels were polarized to different potential values according to their electrochemical behaviors: in the cases of A–P–T and A–P–P, the steels were polarized to the initial passivation potential (marked in Fig. 1a and e); in the case of sP–T, the steels were polarized to the transpassivation potential (marked in Fig. 1b); in the case of sP–P, the steels were polarized to the pitting potential (marked in Fig. 1f); and in the case of A, the steels were polarized to the anodic potential value equal to the pitting potential. After this, SEM was applied to observe the surface morphologies. To facilitate comparison, the detailed results of the SEM observation are also listed in Table 2. Based on the results shown in Table 2, under the electrochemical characteristic of the A–P transition, an obvious corrosion dissolution along the GBs has been observed on the steel surface, indicating that the occurrence of GBD is a general corrosion behavior for the L80, N80 and X65 steels, rather than a special corrosion behavior as in the case of the Q235 steel. Moreover, the presence of non-uniform passive films, mechanical scratches and corrosion pits was observed on the steel surface under the electrochemical behaviors of sP–T, A and sP–P, respectively.

As observed from the abovementioned results, the relationship between the electrochemical characteristic of the A–P transition and the occurrence of GBD for the L80, N80, X65 and Q235 steels in HNO_3_–NaNO_2_, HCl–NaNO_2_ and HAc–NaNO_2_ solutions was very close. The appearance of the A–P transition in the PPC indicated the occurrence of GBD on the steel surface and *vice versa*. However, note that the effects of the acid type, pH value and steel type on the electrochemical behaviors were very significant and have been discussed hereinafter.

For the L80 steel in the pH 2 solutions, the electrochemical behaviors of A–P–T and A were respectively shown in the PPCs in the pH 2 HNO_3_–NaNO_2_ solution and pH 2 HCl–NaNO_2_ solution, which were mainly attributed to the difference in the oxidability and the components HNO_3_ and HCl. HNO_3_ showed both strong oxidability and strong acidity. In contrast, HCl did not show oxidability and strong acidity. Moreover, the inhibition of NO_3_^−^ and the aggression of Cl^−^ in corrosive environments were reported. NO_3_^−^ promoted passivation on the steel surface;^32^ however, Cl^−^ attacked the surface passive film.^33^ Therefore, the presence of the A–P transition in the pH 2 HNO_3_–NaNO_2_ solution and the corresponding absence in the pH 2 HCl–NaNO_2_ solution was observed. Note that the electrochemical behaviors of the four steels in the pH 1 HCl–NaNO_2_ solution were similar to those in the pH 2 HCl–NaNO_2_ solution. Therefore, the PPC and SEM results in the former solution are not provided herein. Because of the relatively weak acidity of HAc, the pH 1 and pH 2 HAc–NaNO_2_ solutions could not be obtained. In the pH 3 and pH 4 solutions, the L80 steel showed the A–P–T, A–P–P and A–P–T behaviors in the HNO_3_–NaNO_2_, HCl–NaNO_2_ and HAc–NaNO_2_ solutions, respectively. The electrochemical characteristic of the A–P transition was due to the effectiveness of NO_2_^−^ and the decrease in the H^+^ concentration;^34^ moreover, the presence of Cl^−^ derived from HCl was responsible for pitting.^30^ In the pH 5 solutions, the lack of the H^+^ concentration contributed to the sP–T behavior in the HNO_3_–NaNO_2_ and HAc–NaNO_2_ solutions and the sP–P behavior in the HCl–NaNO_2_ solution for the L80 steel, which was attributed to the following reaction:^35–37^

6 2Fe^2+^ + 2OH^−^ + 2NO_2_^−^ → 2NO + γ-Fe_2_O_3_ + H_2_O

The effect of the acid type on the electrochemical behavior for the N80, X65 and Q235 steels was similar to that in the case of the L80 steel.

In the HNO_3_–NaNO_2_ solutions, with an increase in the pH value, the L80 steel showed the electrochemical behaviors of A–P–T in the pH 1–pH 4 solutions and sP–T in the pH 5 solution. The relatively wide pH range from pH 1 to pH 4 for the electrochemical characteristic of the A–P transition was attributed to the strong oxidability of HNO_3_ and the inhibition of NO_3_^−^. In contrast, in the HCl–NaNO_2_ and HAc–NaNO_2_ solutions, the electrochemical characteristic of the A–P transition for the L80 steel was present only in the pH 3 and pH 4 solutions due to the component HCl and the weak acidity of HAc, which has been explained in the previous discussion. In the pH 2 HCl–NaNO_2_ solution, the absence of the A–P transition for the L80 steel was attributed to the excess H^+^ concentration^30^ and the aggression of Cl^−^.^33^ Similarly, the effect of the pH value on the electrochemical behavior for the N80, X65 and Q235 steels was similar to that for the L80 steel.

Regarding the effect of the steel type on the electrochemical behavior, note that with the same acid type and pH value, the four steels showed the same electrochemical behavior; this indicated that the composition and alloyed element did not affect the electrochemical characteristic of the A–P transition and the occurrence of GBD. However, the different SEM morphologies were due to the different microstructures of the four steels, which have been studied further.

**Fig. 2:**
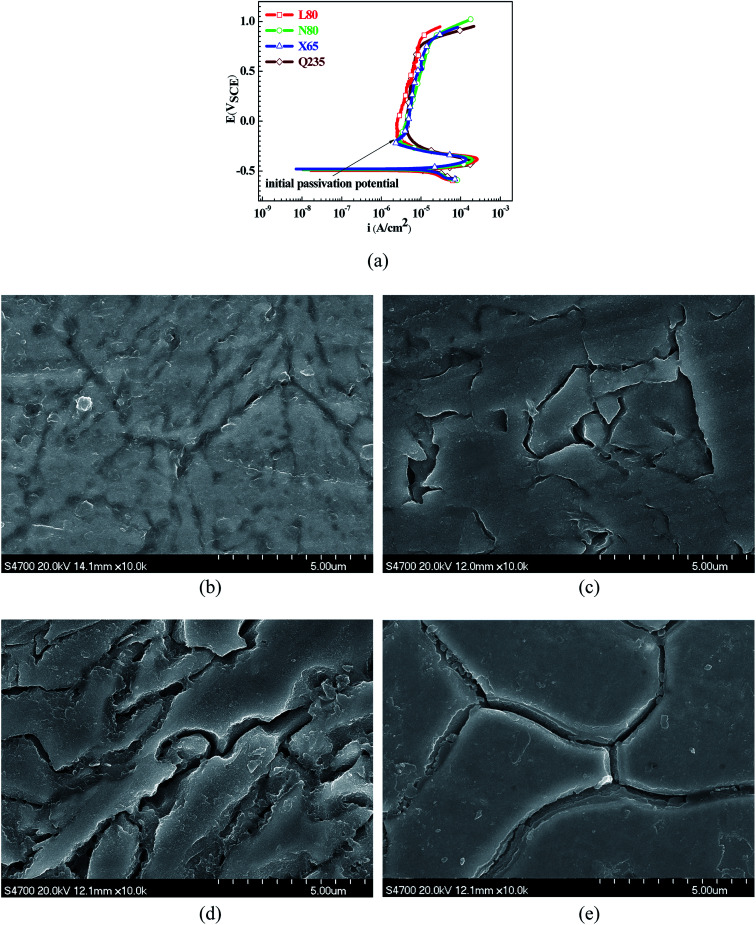
Potentiodynamic polarization curves and surface SEM morphologies of the L80, N80, X65 and Q235 steels in a CO_2_–NaNO_2_ solution: (a) potentiodynamic polarization curves, (b) L80 SEM image, (c) N80 SEM image, (d) X65 SEM image and (e) Q235 SEM image.

To further confirm the relationship between the A–P transition and GBD, the four steels were polarized to the initial passivation potential (marked in Fig. 2a) in the CO_2_–NaNO_2_ solution and then observed by SEM. The results of the PPC test and SEM observation are shown in Fig. 2. All four steels showed the electrochemical behavior of A–P–T, and an obvious GBD was observed on the steel surface. For the L80, N80, X65 and Q235 steels, the results obtained in the CO_2_–NaNO_2_ solution were consistent with those obtained in the HNO_3_–NaNO_2_, HCl–NaNO_2_ and HAc–NaNO_2_ solutions: GBD could be observed on the steel surface at pH 3.7, which was in the range from pH 3 to pH 4.

However, it should be clarified that in a previous study, it has been reported that the occurrence of GBD is attributed to the combined effects of CO_2_ and NO_2_^−^. NO_2_^−^ promoted passivation on the surface of the crystal grains, whereas CO_2_ induced dissolution in the vicinity of the grain boundaries.^27^ In this study, it was concluded that H^+^, rather than CO_2_, resulted in the occurrence of GBD in acidic solutions containing NO_2_^−^. For the L80, N80, X65 and Q235 steels in the HNO_3_–NaNO_2_, HCl–NaNO_2_, HAc–NaNO_2_ and CO_2_–NaNO_2_ solutions, the relationship between the A–P transition and GBD has been discussed hereinafter.

**Fig. 3:**
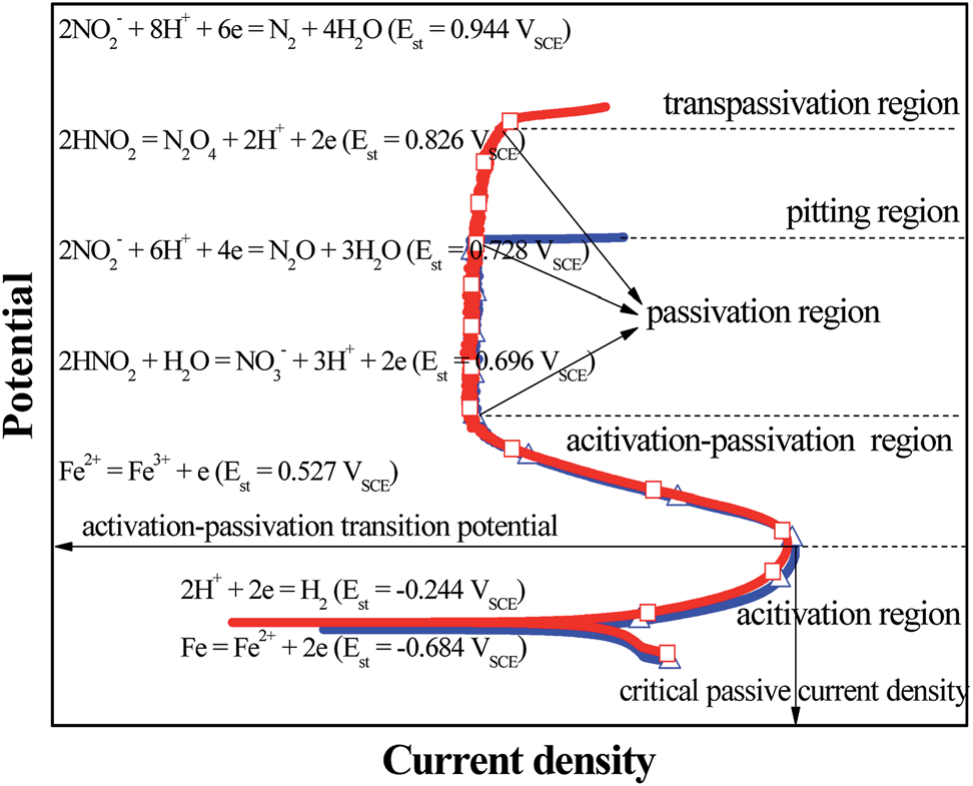
Schematic describing the electrode reactions occurring in the A region and A–P region.

GBD was observed on the steel surface when the four steels showed the electrochemical behaviors of A–P–T and A–P–P. Before the applied potential reached the transpassivation potential and the pitting potential, the PPCs of A–P–T and A–P–P were similar and composed of an activation (A) region, activation–passivation (A–P) region and passivation (P) region. Furthermore, according to previous studies^27–30^ and the current results obtained in this study, the initiation and propagation of GBD occurred before the four steels were polarized in the P region. Therefore, this study was mainly focused on the anodic and cathodic reactions occurring in the A region and A–P region. Fig. 3 shows a schematic to describe the electrode reactions in the A region and the A–P region. For the L80, N80, X65 and Q235 steels in the HNO_3_–NaNO_2_, HCl–NaNO_2_, HAc–NaNO_2_ and CO_2_–NaNO_2_ solutions, before the applied potential reached the A–P transition potential, the dominant anodic and cathodic reactions were Fe oxidation (eqn (5)) and H^+^ reduction (eqn (1)), respectively. With a positive shift of the applied potential, the electrode reactions accelerated. Therefore, the anodic current density gradually increased with potential in the A region until the applied potential reached the A–P transition potential. However, due to the presence of NO_2_^−^ and the continuously positive shift of the applied potential, the following cathodic reactions involving the NO_2_^−^/HNO_2_ reduction occurred:

7 2NO_2_^−^ + 8H^+^ + 6e → N_2_ + 4H_2_O (*E*_st_ = 0.944 V_SCE_)^38^

8 2HNO_2_ → N_2_O_4_ + 2H^+^ + 2e (*E*_st_ = 0.826 V_SCE_)^11^

9 2NO_2_^−^ + 6H^+^ + 4e → N_2_O + 3H_2_O (*E*_st_ = 0.728 V_SCE_)^38^

10 HNO_2_ + H_2_O → NO_3_^−^ + 3H^+^ + 2e (*E*_st_ = 0.696 V_SCE_)^11^

Moreover, the anodic reaction of Fe^2+^ oxidation may be possible:

11 Fe^2+^ → Fe^3+^ + e (*E*_st_ = 0.527 V_SCE_)

The reason is that the *E*_st_ of NO_2_^−^/HNO_2_ reductions (eqn (7)–(10)) is significantly positive than that of the Fe^2+^ oxidation (eqn (11)). However, kinetic studies and potential calculations are necessary to verify the actual cathodic reactions of the NO_2_^−^/HNO_2_ reduction. It is generally accepted that the electrochemical characteristic of the A–P transition is very closely associated with the generation of Fe^3+^, which is the main component in the surface passive film.^39–41^ With a positive shift of the applied potential, when the applied potential reached up to the A–P transition potential, the anodic current density gradually decreased with potential in the A–P region; this suggested the activation of Fe^2+^ oxidation and the formation of the passive film. Furthermore, the cathodic reactions for the NO_2_^−^/HNO_2_ reduction played a critical role in the abovementioned process.^28–31^ On the other hand, the metallurgical phenomenon of the grain boundary segregation (GBS) was generally present on the alloy steels^42–44^ and carbon steels,^45–47^ which resulted in the difference between passivation capabilities of GIs and GBs.^48–50^ In a previous study, it has been reported that GIs containing high level of Fe showed better passivation capability than GBs with high levels of Si and Mn.^28^ Therefore, when the applied potential moved to the A–P region, the electrochemical micro-couple between the GIs and GBs was established spontaneously to induce the occurrence of GBD. However, between the GIs and the GBs, the detailed mechanisms about the establishment of the electrochemical micro-couple and the driving force for the potential difference need further investigation.

## 4. Conclusions

In this study, four steels (L80, N80, X65 and Q235) were investigated in four acidic solutions (HNO_3_, HCl, HAc and CO_2_) containing NO_2_^−^ to reveal the relationship between the electrochemical characteristic of the A–P transition and the occurrence of GBD. The main conclusions were as follows:

(1) The relationship between the A–P transition and GBD was very close. The appearance of the A–P transition in the PPC indicated the occurrence of GBD on the steel surface. Moreover, it was confirmed that under the electrochemical characteristic of the A–P transition, the occurrence of GBD was a general corrosion behavior for carbon steels and alloy steels in acidic media containing NO_2_^−^.

(2) The effect of the steel type on the electrochemical characteristic of the A–P transition and the occurrence of GBD was not obvious. However, the effects of acid type and pH value were very significant. The A–P transition and the GBD were present from pH 1 to pH 4 in the HNO_3_–NaNO_2_ solutions, at pH 3 and pH 4 in the HCl–NaNO_2_ solutions and at pH 3 and pH 4 in the HAc–NaNO_2_ solutions.

## Conflicts of interest

There are no conflicts to declare.

## Supplementary Material
